# EUPID—configurable privacy-preserving record linkage in federated health data spaces

**DOI:** 10.3389/fdgth.2026.1751234

**Published:** 2026-02-09

**Authors:** Dieter Hayn, Emanuel Sandner, Martin Baumgartner, Bernhard Jammerbund, Fabian Wiesmüller, Stefan Beyer, Hannah Vinatzer, Angelika Rzepka, Klaus Donsa, Karl Kreiner, Guenter Schreier

**Affiliations:** 1 Digital Health Information Systems, Center for Health and Bioresources, AIT Austrian Institute of Technology, Graz, Austria; 2 Department for Computer Science and Biomedical Engineering, Institute of Neural Engineering, Technical University Graz, Graz, Austria

**Keywords:** European Health Data Space (EHDS), findability accessibility interoperability re-usability (FAIR), privacy-preservation, record linkage, secondary use

## Abstract

**Introduction:**

Rare disease research relies heavily on secondary use of health data due to the scarcity of clinical guidelines and data sharing between research institutions and hospitals. Linking rare disease patients is challenging due to increased re-identification risk in small cohorts, thus limiting the data's potential for research. Privacy-Preserving Record Linkage (PPRL) enables the linkage of disparate datasets while safeguarding the identities of involved participants.

**Methods:**

The aim of the present paper is to provide an up-to-date description of the concept and the technical details of the European Patient Identity (EUPID) Services, a configurable PPRL solution which is currently used for rare disease research in Europe to bridge healthcare and research. They support different algorithms for record linkage (configurable selection of quasi-identifiers, various hashing algorithms, phonetic hashing, Bloom filters), re-identification and flexible specification of the pseudonym format. Furthermore, their setup is also flexible whether to install standalone instances or integrate with a central EUPID Services deployment.

**Results:**

The EUPID Services have been used in various research applications since 2014. As of July 2025, 6,356 unique patients have been registered to the central EUPID Services within the domain Paediatric Oncology in Europe, and 10,340 pseudonyms for 12 EUPID Contexts have been generated. Within the Austrian Health Data Donation Space, which represents a federated PPRL infrastructure supporting asynchronous record linkage, more than 16 million patients were pseudonymised in six different contexts. Overall, four cases of false negative matches have been identified, which were caused by typing errors. So far, no false positive match has ever been detected.

**Discussion:**

In view of the upcoming European legislatives like the European Health Data Space (EHDS), connecting patient data securely and safely will become increasingly important and useful. The EUPID Services support such linkage in a Findable, Accessible, Interoperable and Reusable (FAIR) manner and thus could represent a vital and proven part of future national and European research networks.

## 1 Introduction

Healthcare data that were collected for a specific purpose (primary use) represent a valuable resource for further research. Such “secondary use” of healthcare data often faces barriers due to data protection concerns, intellectual property rights, and technical challenges due to heterogenous and distributed data. This applies especially for rare diseases, where the limited data available are spread world-wide and, in the absence of clinical guidelines, patients are typically enrolled to clinical trials and treated according to the most recent trial protocols (1). Additionally, rare disease data are often distributed over different types of resources, such as clinical trial databases, biobanks, and registries. While routine care diagnosis and treatment data are collected in a personalised format, research data are typically pseudonymised to comply with the General Data Protection Regulation (GDPR) (2).

Secondary use of distributed healthcare data is currently addressed by various ongoing initiatives. In alignment with the current regulations for sharing research data (3), the European Commission has established the European Health Data Space (EHDS), with the aim to improve health data usage in the European Union (EU). Many of these activities aim at data provision in a Findable, Accessible, Interoperable and Reusable [FAIR (4)], manner, such as the European Rare Diseases Research Alliance (ERDERA) (3).

Privacy-Preserving Record Linkage (PPRL) concerns the linkage of different datasets without disclosing the participant's Personally Identifiable Information (PII), by applying different algorithms on so-called quasi-identifiers (QIDs), i.e., parameter sets than can be used to identify a patient in a unique way.

PPRL addresses a wide range of application scenarios. For paediatric oncology, related use cases have been summarised in a prior study (5), based on six dimensions: distributed personalised records, pseudonymisation, distributed pseudonymised records, record linkage, storage, and analysis. The importance of PPRL in rare disease research has recently been further highlighted in a Lancet Oncology comment, where the European Society for Paediatric Oncology (SIOP) formulated six recommendations for improving the current legislative framework, one of them, recommendation four, focusing on the “support for privacy-preserving data linkage” (6). Already in 2013, to better understand the differences between PPRL solutions, Vatsalan et al. published a PPRL taxonomy, listing fifteen dimensions that can be used to characterise privacy-preserving record linkage techniques (7). More recently in 2021, Gkoulalas-Divanis et al. published a review article comparing modern PPRL techniques and systems using a taxonomy consisting of the four aspect “families”: computation, utility, privacy, and practical aspects (8). A comprehensive overview of various aspects of PPRL was published by Christen et al. (9).

**Figure 1:**
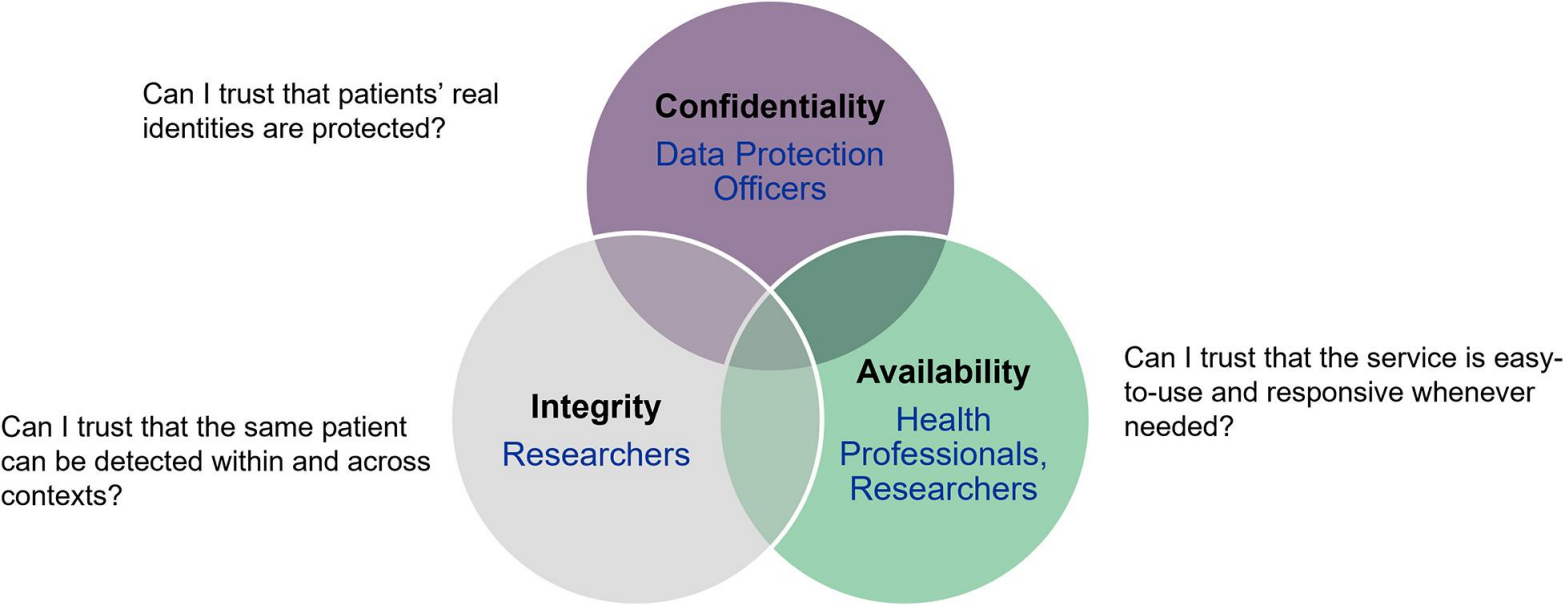
Confidentiality-Integrity-Availability (CIA) triad—basic law of information security, adapted to the privacy-preserving record linkage context.

State-of-the-art PPRL algorithms can be based on phonetic coding (10, 11), hashing (12, 13), reference-values (14, 15), embedding (16–18), differential privacy (19), or secure multiparty computation (applied on QIDs or on the clinical data themselves) (20–23). Some solutions only support linkage in case of perfectly matching records. More comprehensive solutions can also link slightly differing records, e.g., in case of typing errors or missing data. Since both—false positive as well as false negative linkage—may have severe consequences, the optimal threshold of accordance must be chosen depending on the use case. This problem is related to the basic law of Information Security, the Confidentiality-Integrity-Availability (CIA) Triad (24), as illustrated in Figure 1.

There are various PPRL services on the market, such as (in alphabetic order) AnonLink (25), CRID (26), E-PIX (27), FEMRL (28), FRIL (29), GRHANITE (30), LinkWise (31), LinXmart (32), LSHDB (33), MainSEL (22), Mainzelliste (34), MERLIN (35), MTB (36), NGLMS (37), NIH GUID (38), OneFL Deduper (39), P4Join (40), PRIVATEER (41), SOEMPI (42), SPIDER1, TAILOR (43). Many of these solutions have successfully been applied in selected application scenarios, proving feasibility of PPRL in various settings. However, there is a need for PPRL services which support multiple scenarios as described below, with configurable working points based on the CIA Triad, and which is also mature and sustainable enough to be applied even in long-term secondary use scenarios.

The European Patient Identity (EUPID) Services represent a hash-based PPRL solution, which has initially been described in 2014 (44). They are widely used in rare disease research in Europe. The EUPID Services are operated by the AIT Austrian Institute of Technology, which acts as a data processor on behalf of data controllers holding a EUPID license. The services are financed either through funded research, such as national or international research projects, or through contract research. Although initially developed for European pediatric oncology projects, the EUPID Services have, over the time, proven to be a valuable tool for a broader range of application areas, including national research infrastructures and temporary research projects. Since the EUPID Services have last been described in 2014, the underlying algorithms and principles have changed significantly.

The aim of the present paper is to provide an up-to-date description of the concept and the technical details of the EUPID Services, a configurable PPRL solution which is currently used for rare disease research in Europe, and in various research projects with distributed data sources. In addition, accuracy, efficacy and implementation status should be analysed. Based on the EUPID Services' implementation and specifications, PPRL providers and stakeholders in need of PPRL solutions will be informed on how to successfully apply PPRL in various scenarios.

## 2 Methods

### 2.1 EUPID services concept

**Figure 2:**
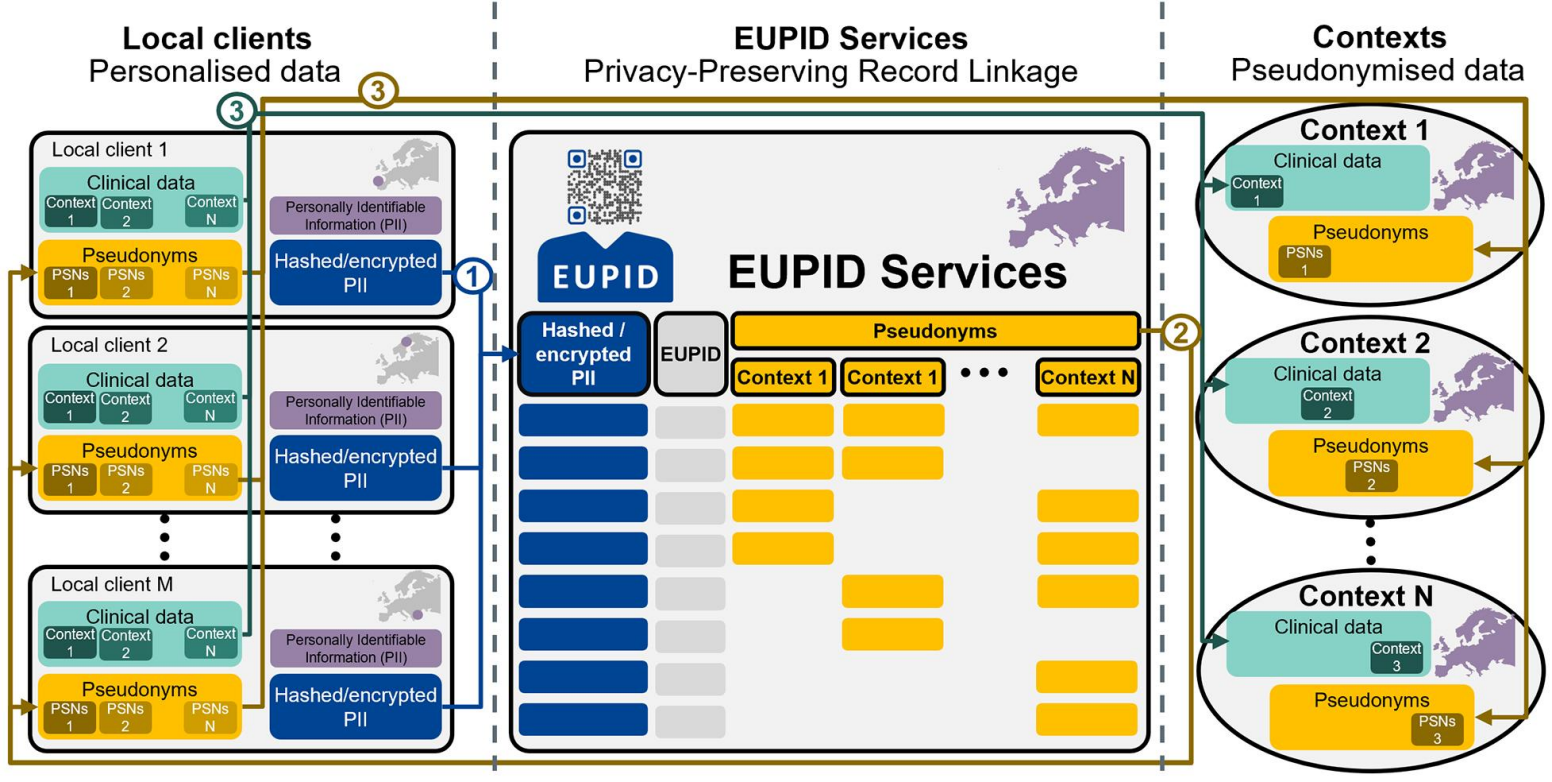
The EUPID concept separates local clients (left), holding personally identifiable information (PII), from the EUPID services (middle), which ensure linkability, and the contexts (right), who hold pseudonymised, aggregated data. The patient registration process consists of: 1) Local clients hash and/or encrypt PIIs of their patients and send the encrypted and/or hashed PII to the EUPID Services. 2) The EUPID Services link the PII from different local clients and different context to the unique EUPID and return context-specific pseudonyms to the local clients. 3) The local clients send the context-specific clinical data together with the context-specific pseudonyms to the respective contexts. Maps created using MapChart (https://www.mapchart.net/), licensed under CC BY-SA 4.0. “EUPID” logo reproduced with permission from European Patient Identity Services (https://services.eupid.eu/).

The EUPID Services concept (Figure 2) is based on the separation of a) clients (e.g., in hospitals) who hold personalised clinical data of their own patients, b) context providers (e.g., for registries or clinical trials), who hold pseudonymised clinical data from multiple centres, and c) encrypted and/or hashed data derived from QIDs which refer to context-specific pseudonyms and which are used for the actual record linkage.

The EUPID Services do not require any installation of software at the computers of local clients. Only a standard internet browser is required. Specific software for communication with the EUPID Application Programming Interface (API) only needs to be included at the data collection service of each context (e.g., JavaScript within electronic data capture systems).

### 2.2 EUPID services IT architecture

**Figure 3:**
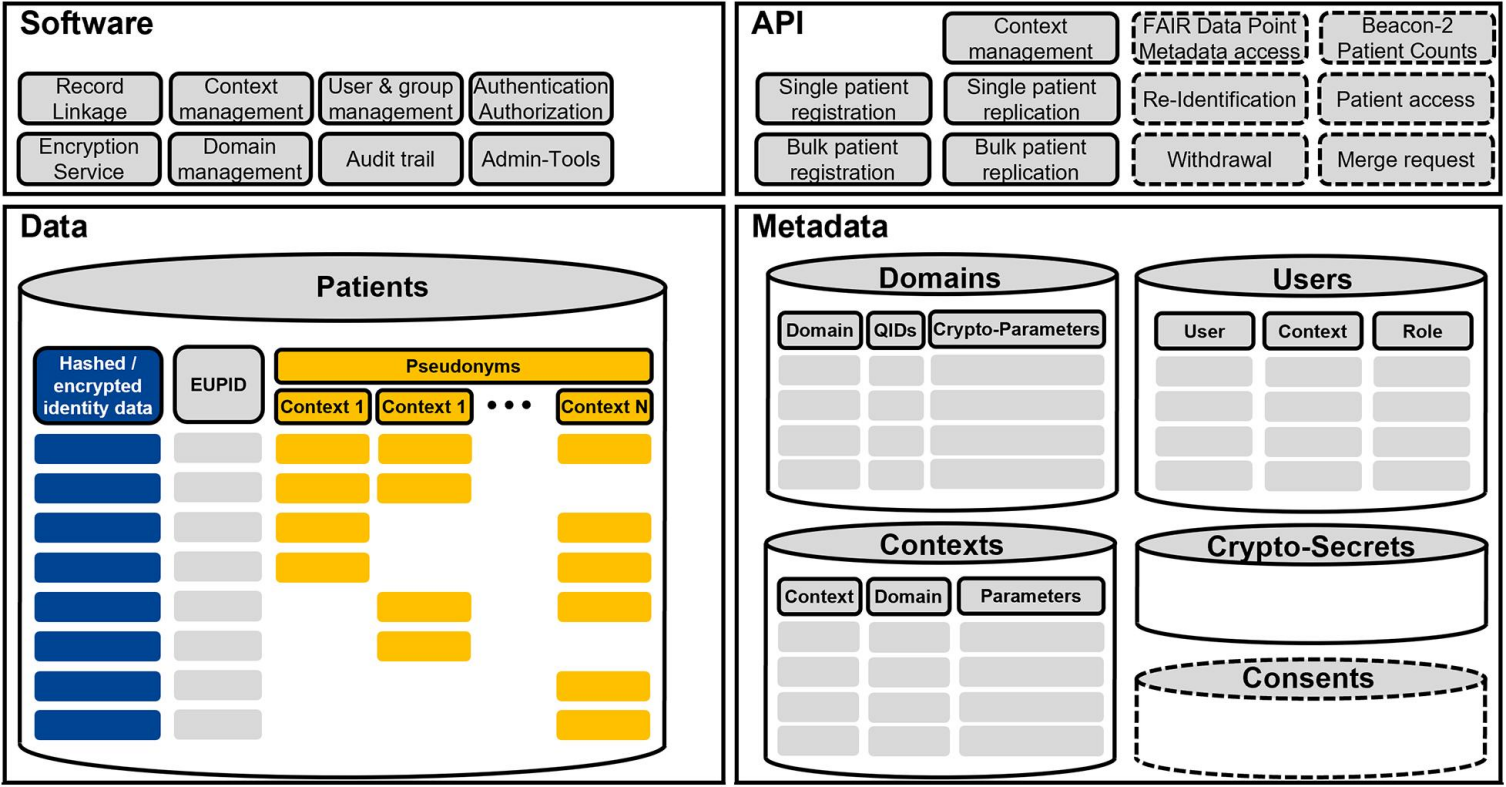
EUPID services IT architecture, consisting of software modules, an API, the actual PPRL data, and metadata. Dashed sections are foreseen but not implemented yet.

The EUPID Services consist of specific software and a dedicated database for data and metadata. The infrastructure is accessible via a Web API to an arbitrary number of consumer applications within client infrastructures. All data are symmetrically encrypted prior storage in the database. Figure 3 provides an overview of the overall architecture.

#### 2.2.1 EUPID services data

The EUPID patient database represents the core for all PPRL applications within the EUPID Services. Within the patient database, each patient is assigned a EUPID, which represents a Universally Unique Identifier (UUID). This EUPID refers to a) hashed PIIs which are used for PPRL, b) context specific pseudonyms, which are provided to the EUPID users, and optionally c) encrypted PIIs.

All EUPID data are stored using symmetric encryption at REST within the EUPID Database, with the encryption key stored outside of the database. Only pseudonyms are communicated to external services, while the EUPIDs themselves as well as hashed and/or encrypted data never leave the EUPID Services infrastructure.

#### 2.2.2 EUPID services metadata

The EUPID Services support PPRL within a specific EUPID Domain, such as “Paediatric Oncology in Europe” or “Austrian Health Research Infrastructure”. Data can however not be linked between different domains. For each EUPID Domain, the used QIDs, cryptographic algorithms, respective parameters and secrets are specified.

Each EUPID Domain contains several EUPID Contexts, such as registries, clinical trials, biobanks, etc. Each of these contexts hold different pseudonyms for individual patients, which can be linked via the EUPID Services. For each context, additional parameters, such as URLs, contacts and principal investigator information, etc., can be provided.

Two types of EUPID Users are supported. So called Managed Users are managed by the EUPID Services Provider and stored within the EUPID Services IT infrastructure. Managed Users can have different roles in different contexts. Delegated Users are stored by EUPID Context Providers, such as the provider of a registry. If specific security requirements are met, the EUPID Services will trust that users accessing the EUPID API from a trusted EUPID Context Provider are authenticated to access the EUPID API as delegated users.

It is foreseen that consents and use conditions will be stored within the EUPID Services metadata. However, so far these data are handled document-based, outside the EUPID Services infrastructure.

#### 2.2.3 EUPID services software

The actual record linkage is done by the EUPID Services software, as described in section 2.3 and for managing communication between the EUPID database and other components. In addition, the software ensures authorization and authentication of users accessing the service, audit trailing, user and group management, and domain and context management. Authorization, authentication and user management can either be implemented locally on the EUPID Server, or external services such as Azure Entra ID or any other OAuth 2.0 compliant identity provider can be used.

#### 2.2.4 EUPID services API

External services can access the EUPID Services via an API. Every request requires an OAuth 2.0 Bearer Token2 to be sent in the authorization header.

The API supports the following management requests:

- Get contexts—Retrieve a list of available contexts within a domain
- Add context—Add a new context to a domain
- Assign context to user—Assign a context to a user to enable them to register patients in that context.
- List context assignments—Retrieve a list of all contexts assigned to a specific user.
- Remove context from user—Remove the assignment of a context from a specific user. The user can no longer register patients in that context.

The following API endpoints to register a single patient are available:

- Initialisation—Provides a list of contexts where the user has permission to register patients, including information on supported QIDs and cryptographic algorithms and parameters as described below.
- Register patient—Register a new patient based on the ContextID of the target context and the patient's hashed QIDs. If specified for the target context, additional encrypted QIDs can be provided. An optional clarification parameter can be included in the request to specify whether patients should be linked after a partial match is detected in a preceding API-call (see section 2.3.2). The endpoint returns a patient registration response code which describes the result of the registration process (such as “No match found—a new pseudonym was generated” or “Full match in another context found”). If the patient was successfully registered, the response also includes the patient pseudonym for the context. If a partial match requires user interaction, the endpoint additionally returns a clarification code from which the user must select the appropriate option to conclude the registration process.
- Replicate patient—Register a patient in a target context, based on their pre-existing pseudonym in a source context.

Additionally, sets of patients can be registered via the bulk registration API endpoints:

- Bulk registration of multiple patients—Initiate registration of multiple patients at once. As payload, a list of patient registration records (each following the format for single patient registration described above) is provided. Additionally, each patient record must include a unique PatientReference. The registration process is handled asynchronously. The endpoint returns a unique bulk registration ID. The number of patients which can be registered at once can be limited to avoid overloading the EUPID Services instance.
- Show all previous bulk registration requests—Returns the bulk registration set IDs of all previous bulk registration sets for the user of the provided Bearer Token.
- Show bulk registration results—Returns the registration results of all patient registration records for a given bulk registration set ID. The response contains the creation date, creating user, a status indicator on whether the bulk registration request is being processed, a progress value indicating to which extend the bulk registration task is already finished, and a finished date. Additionally, an array of RegistrationResponses is provided, which includes information for each patient, corresponding to the single patient registration results.

### 2.3 Real-time privacy-preserving record linkage

#### 2.3.1 Domain-specific (quasi-) identifiers

To support PPRL within a EUPID Domain, specific parameters—either unique identifiers (IDs) or sets of QIDs—are specified. Unique IDs can be social insurance numbers, patient IDs, etc. Sets of QIDs can be first name & last name & date of birth, etc. For each domain, one or more supported IDs and sets of QIDs are specified, depending on the availability of data and on the linkage requirements (e.g., in rare disease research, first name, last name and date of birth may be sufficient to identify patients with acceptable accuracy, while additional QIDs such as place of birth might be required for national research infrastructures). Each patient-registering EUPID Context within the EUPID Domain must provide at least one of these IDs or sets of QIDs. In addition, the EUPID Services support non-patient registering EUPID Contexts, whose patients and pseudonyms can only be derived by replicating patients from other EUPID Contexts, i.e., deriving a new pseudonym for a pre-existing patient in another EUPID Context.

#### 2.3.2 Domain-specific cryptographic algorithms

The EUPID Services support different kinds of algorithms which can be applied for PPRL in a specific EUPID Domain. Before these algorithms are applied on the EUPID Domain's QIDs, textual QIDs such as first name or last name are normalised with the following steps:

1. Conversion to lower case
2. Conversion of characters NOT part of the Unicode categories L* (Letters), N* (Numbers), and Co (Private use) to spaces.
3. Application of Unicode normalization to decompose accented characters into their base and diacritic parts (NFKD form), and subsequent removal of the diacritic marks
4. Removal of trailing and leading spaces

Date-based QIDs (such as the date of birth) are trimmed to remove trailing and leading spaces from the input string. Dates must be provided in ISO-8601-compliat format yyyy-mm-dd.

For security reasons, PPRL is done on a record-level, where each record is composed of certain QIDs. Therefore, all QIDs of a record are concatenated to a single string, a EUPID Domain-specific cryptographic salt can optionally be added, and the respective cryptographic algorithm is applied on the result.

Real-time PPRL is based on hashing, phonetic hashing or Bloom filtering of QIDs. Phonetic hashing or Bloom filters ensure that PPRL is possible even in case of different spellings of names (“Müller” vs. “Mueller”).

The following cryptographic algorithms are currently supported:

- Hashing
  - MD5 (45) (has been supported in earlier versions of EUPID and has meanwhile been removed for security reasons)
  - Keying Hash Functions for Message Authentication (HMAC) (46)
  - Argon2 (47)
- Phonetic hashing
  - Cologne (48)
  - Soundex (49)
- Bloom-filters (50)

For each cryptographic algorithm, specific parameters need to be defined per EUPID Domain. Examples of parameters include the number of MD5 hashing cycles for MD5. For Argon2, memory cost (defines the memory usage), time cost (defines the amount of computation realised and therefore the execution time), parallelism (defines the number of parallel threads), hash length (defines the length of the resulting hash), type (defines the Argon2 variant to use) can be specified. Bloom-filters support different bit array sizes and numbers of hash functions. Additionally, Bloom-filters can be configured to apply Cryptographic Longterm Keys (51), geo-coded representation of places (52), and locality-sensitive hashing (53).

Each EUPID Domain can be based on one or more combinations of QIDs and cryptographic algorithms, which can each be specified as fully or partially matching. Linkage algorithms specified as full matching lead to immediate linkage of data, whereas in case of partial matches, manual decisions are required. Therefore, the API returns a case-specific response and clarification code which indicate the partially matching QID. The user can check whether potential typing errors occurred and re-send the request, including the respective clarification code (see section 2.2.4).

#### 2.3.3 Re-identification

In addition to record linkage, as an independent service, the EUPID Services optionally support re-identification of patients based on asymmetric encryption, whereas a public key is used by the local sites to encrypt identity data while the respective private key is kept secretly by a trusted third party (TTP). Re-identification is supported on different levels:

- Domain-specific re-identification
- Context-specific re-identification
- Site-specific re-identification (e.g., to de-crypt all patients of a specific site, so that the site's own patients can be shown in a personalised rather than pseudonymised way. The private key might be stored within the local site's IT infrastructure)
- Patient-specific re-identification (e.g., to provide an overview, which contexts are currently holding pseudonymised data of the respective patient. The private key might be stored in a patient app)

In any case, a specific TTP is nominated by the owner of the respective level. The TTP generates a cryptographic key pair, publishes the public key and stores the private key in a secure manner, such as in an Azure KeyVault3 or a physical Hardware Security Module (HSM). For any patient registered in the respective level, the client not only calculates the cryptographic elements required for PPRL (e.g., hashes) but also encrypts the QIDs with all relevant public keys. The EUPID Services store all encrypted QIDs.

#### 2.3.4 Pseudonym generation

Context-specific pseudonyms are alphanumeric (2–9, A–Z), randomly generated, 8 characters strings which must be unique within each context. Purely numeric pseudonyms and pseudonyms which are numeric except for the letter “E” are prevented to avoid misinterpretation as numerical instead of textual data by data analysis tools (e.g., Microsoft Excel). To avoid errors due to similarly looking characters, the characters “0” and “O”, as well as “1”, “l” and “I” are not used.

Context-specific prefixes can be defined for each context. A prefix consists of at least three alphanumeric characters (0–9, A–Z), starting with a letter (not a number). Even if a prefix is defined, the pseudonym is stored without prefix in the EUPID patient database. However, the EUPID Services API adds the prefix followed by the character “-” to the EUPID pseudonym when preparing the response.

**Figure 4:**
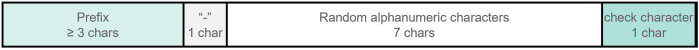
EUPID services pseudonym format including optional prefix and check character.

To support validation of EUPID pseudonyms, the last of the eight characters of the pseudonym is an alphanumeric check character, which is calculated from prefix and the remaining seven characters, using a standardised check character system based on ISO/IEC 7064:20034. The EUPID pseudonym format is illustrated in Figure 4. “ONC-A7ST542G” serves as an illustrative example of a pseudonym generated by the system for a EUPID Context with prefix “ONC”.

### 2.4 Deployment options

**Figure 5:**
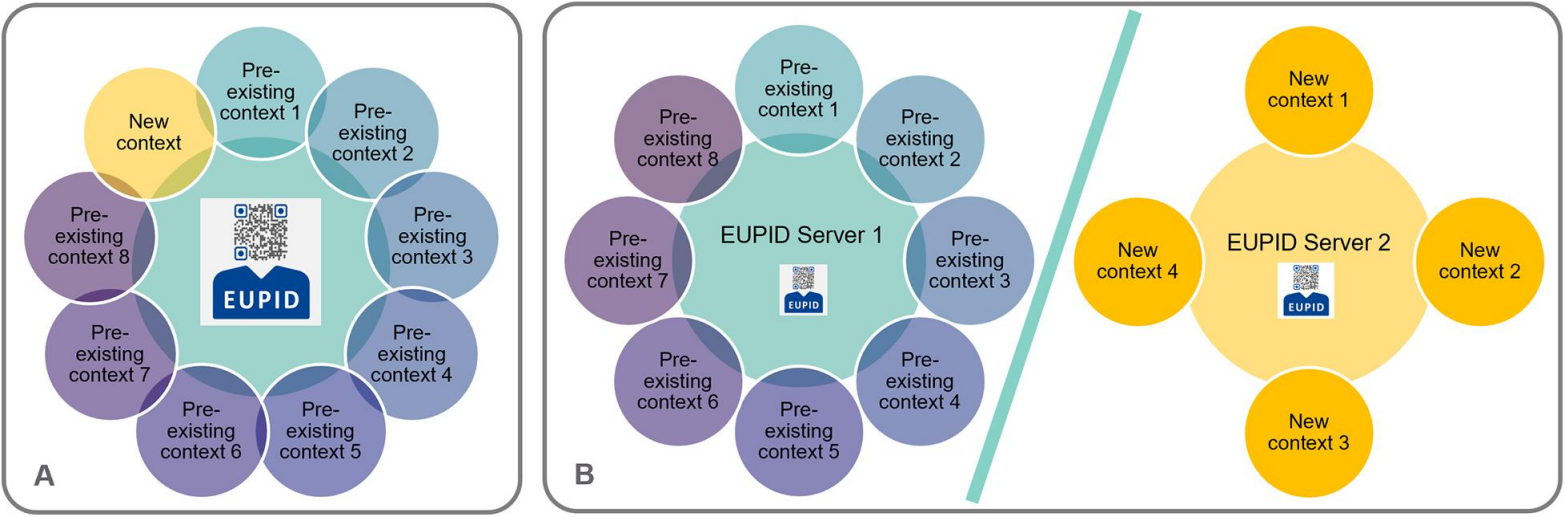
Deployment options—**(A)** A new context can either be added to the EUPID services within the pre-existing EUPID deployment, or **(B)** A separate deployment of the EUPID services can be set up. “EUPID” logo reproduced with permission from European Patient Identity Services (https://services.eupid.eu/).

For a researcher who requires a PPRL service for a specific research question, a research project, a research infrastructure, etc., the EUPID Services can be used in two different deployment settings (see Figure 5):

- Option A—Take use of a pre-existing EUPID deployment and add a new EUPID Context and/or EUPID Domain to this deployment
- Option B—Deploy a separate instance of the EUPID Services and set up a new EUPID Domain. The separate instance can be deployed in the cloud, on the servers of a EUPID Provider, or on a server of the researcher.

**Figure 6:**
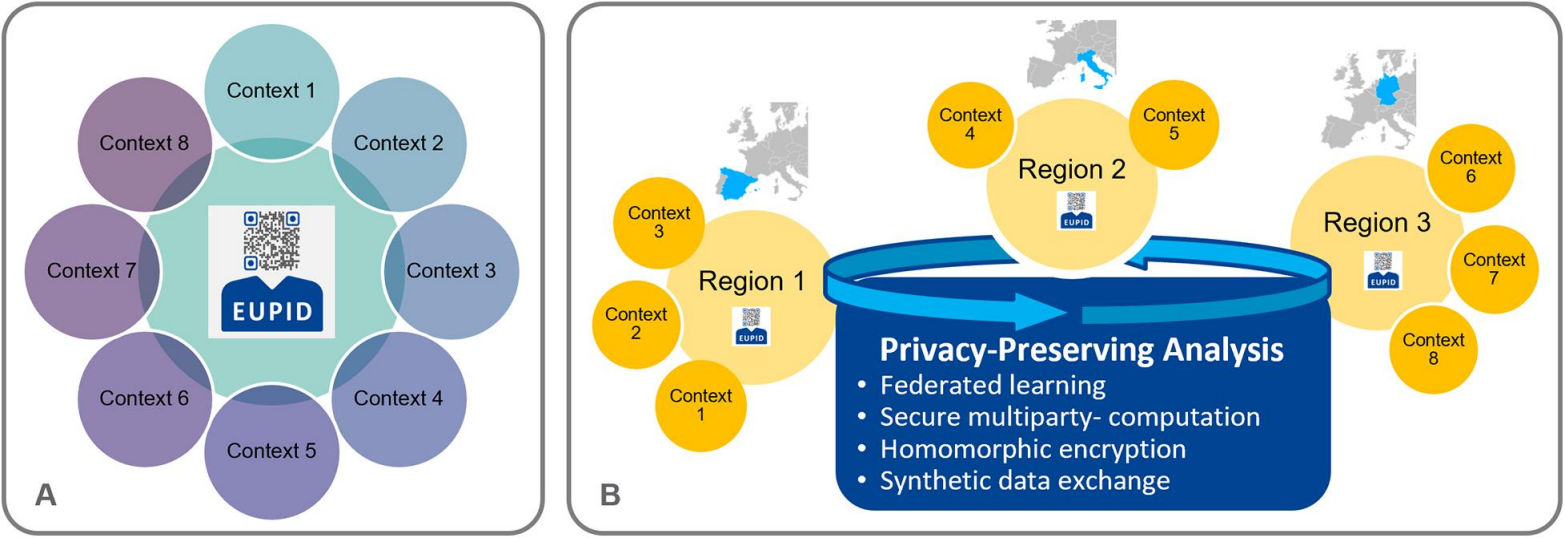
Deployment options for the EUPID services. **(A)** One single central EUPID Service is deployed. All record linkage is done in real-time whenever a patient is registered. **(B)** More than one EUPID Services are deployed e.g., in different geographical regions. Each EUPID Service supports real-time record linkage for sub-sets of contexts. Record linkage between different EUPID Services is done in a non-real-time manner by applying different privacy-preserving analysis methods across regions. Maps created using MapChart (https://www.mapchart.net/), licensed under CC BY-SA 4.0. “EUPID” logo reproduced with permission from European Patient Identity Services (https://services.eupid.eu/).

In addition, as illustrated in Figure 6, the EUPID Services can be operated either based on a central infrastructure (A), or in a distributed setting (B), with different EUPID deployments operated e.g., for different geographic regions or for different context groups.

### 2.5 Asynchronous privacy-preserving record linkage

In setting B described in Figure 6, PPRL between the distributed EUPID Services deployments can be done in various ways, which can be specified depending on the respective application's requirements:

- Provision of all services' hashed/encrypted PIIs to one of the distributed EUPID Services
- Setup of another central EUPID Service which performs the PPRL in between the distributed EUPID Services
- Application of homomorphic encryption and/or secure multiparty computation on the distributed hashed/encrypted PIIs to identify duplicate records in both instances (see e.g., the secure multiparty computation approach by Laud and Pankova 49).

While the first two of these three options have already been implemented, secure multiparty computation/homomorphic encryption has been conceptualised but not implemented yet.

## 3 Results

### 3.1 Use cases

#### 3.1.1 Use case 1—paediatric oncology Europe

In paediatric oncology, the EUPID Services are deployed based on setting A (one central EUPID Service) as shown in Figure 5. All services are deployed in a European Azure cloud. Full matches are either achieved by Argon2 matched sets of first name, last name and date of birth, or by Argon2 matched unique patient IDs. Partial matches are achieved by matched phonetic hashes of first or last names or by flipped day and month of birth. Re-identification is supported for some contexts in the domain, with the European Society for Paediatric Oncology (SIOPE) acting as a TTP. This EUPID Domain is currently being integrated into the ERDERA Virtual Platform by providing metadata concerning the established contexts and counts of patients per context via a Fair Data Point and a Beacon-2-API (54).

The EUPID Services are used in various paediatric oncology applications since 2014. As of July 2025, 6,356 unique patients and 10,340 pseudonyms have been generated within 12 EUPID Contexts within this domain. Record linkage was applied between up to four EUPID Contexts per patient. Table 1 summarises the number of patients depending on the number of contexts the respective patients are registered to.

**Table 1:** Number of patients in the EUPID domain “paediatric oncology” depending on the number of contexts they are registered to.

Number of contexts per patient	Number of patients
1	3,466
2	2,162
3	362
4	366
Total	6,356

EUPID-based PPRL was applied in several use cases in the past years:

- Linking biobanking and clinical data (55, 56)
- Linking clinical data with genomic/phenotypic analysis data (57)
- Linking neurological and oncological trial data (58)
- Artificial intelligence based on linked imaging, clinical and biological data (59, 60)
- A systematic overview of use cases for PPRL in paediatric oncology, from patient counts over tumour board to AI applications, is summarised in (61).

#### 3.1.2 Use case 2—Austrian health research infrastructure

Within the Smart FOX project, an Austrian Health Data Donation Space (AHDDS) is currently being explored (62). Within the AHDDS domain, a decentralised EUPID infrastructure as illustrated in Figure 6B has been set up, with three different stakeholders [regional hospital provider Tirol Kliniken, based on the infrastructure described in (63), Medical University of Graz, IT services of the Austrian social insurances], each operating their own EUPID instance on their local servers. All three instances share the same cryptographic algorithms and parameters. Asynchronous linkage is applied semi-automatically on demand, only for specific research questions, which each require a separate ethics approval.

Within the AHDDS, full matches are applied on Argon2 matched patient IDs. Partial matches are achieved based on sets of first names, last names, and dates of birth.

As of July 2025, more than 16 million unique patients have been generated within 6 EUPID Contexts in this domain.

### 3.2 Confidentiality, integrity, and availability

Based on the CIA Triad (24), any IT application needs to find the right balance between confidentiality, integrity and availability. In the following, these three aspects are described in more detail based on selected working points of the implementation areas described above.

#### 3.2.1 Confidentiality/privacy & security

The security concept of the EUPID Services has been audited and confirmed to be secure by two independent IT security consulting companies in Europe. No security breach has been identified so far. As of January 2026, GDPR conformity certification by an accredited GDPR certification body is in the final stages.

#### 3.2.2 Integrity/linkage accuracy

In their current application areas, the EUPID Services have been set up for use cases that require low false positive rates, while accepting the risk of isolated false negative cases. By combining the strict linkage properties of hashed PIIs (full matches) with phonetic hashing in the partial match workflow, the current setup proved suitable for the scenarios addressed so far. In the PRIMAGE project (59, 60), we have identified four false negative matches, which were all due to typing errors when entering unique patient IDs. So far, no false positive match has been identified.

#### 3.2.3 Availability/usability & performance

Based on a variety of cryptographic algorithms, the EUPID Services support different working points, ensuring the right balance between confidentiality, integrity and availability of data for each application scenario. In paediatric oncology in Europe, we use first name, last name and date of birth as QIDs, Argon2 hashes of those QIDs for full matches, Soundex and Cologne phonetic hashes applied on first name and last name as well as flipped day and month of birth for partial matches. In this setting, preparation of a patient registration call to the EUPID API, which includes calculating five Argon2 hashes, takes approximately 40 ms per hash, i.e., 200 ms in total on a workstation with an Intel Xeon W-2145 CPU @ 3.7 Ghz and 32 GB RAM. The hash was calculated in JavaScript and run in the Brave browser. However, the actual hashing performance highly depends on the available hardware specification and used software and execution environment.

For onboarding new contexts to the EUPID Services, the following dedicated onboarding process has been established:

- Provide detailed specification to the context provider
- Context provider implements the API interface and connects to the EUPID test services
- Test implementation based on a pre-defined test protocol, ensuring that all cryptographic measures have been implemented correctly by the context provider
- Deploy on productive EUPID Services

For EUPID Client providers, onboarding typically takes approximately two to four person weeks.

### 3.3 Legal and policy aspects

Legal pre-requisites for linking patient data are confirmed by the context owners, typically based on data processing agreements and/or licensing agreements between the EUPID Provider and the EUPID Context Owner. Typically, usage of the EUPID Services for pseudonymisation and PPRL is described in the study protocol and included in the informed consent which are approved by the research project's ethics committee. Data controllers using the EUPID Services are responsible for securely storing the signed informed consent forms. Any linkage with new EUPID Contexts must be explicitly approved by the data controller. The EUPID Terms of Use specify how the EUPID Services and the related information (e.g., context specific pseudonyms) may be used. When registering new patients, users confirm that they read and accept the EUPID Terms of Use and that they have the right to register patients to the EUPID Services. Policy documents for EUPID Domain Owners and TTPs have been set up.

## 4 Discussion

This paper presents the technical and procedural implementation of the EUPID Services, a PPRL infrastructure that has been successfully deployed in domains such as rare diseases, paediatric oncology, and national health research initiatives in Austria. The described setup demonstrates a flexible, standards-compliant approach that can serve as a blueprint for future PPRL solutions, especially in regulated health data environments.

Our findings highlight that there is no “one-size-fits-all” solution for PPRL. Even within a narrowly defined domain such as pediatric oncology, a wide range of use cases exists, each with different requirements concerning linkage precision, data availability, security, and consent.

The EUPID Services respond to this diversity by allowing a tailored setup for each EUPID Domain, balancing confidentiality, integrity, and availability in line with the CIA triad. This modular and configurable architecture is of particular value for both long-term research infrastructures and temporary, project-based data collaborations. The success of EUPID in these contexts demonstrates its utility in supporting scalable, GDPR-compliant PPRL across diverse scenarios.

While many technical solutions for PPRL already exist in the broader ecosystem, EUPID contributes additional capabilities to this landscape. In particular, it complements existing solutions by offering interoperability with established infrastructures and standards. This has enabled its integration into prominent European research platforms such as the European Joint Programme on Rare Diseases (EJP RD) Virtual Platform (54), GPAP (57), and the ESCP registry of the European Reference Network for Paediatric Cancer (64), among others.

Despite the demonstrated flexibility and reliability of the EUPID Services, several limitations remain. As with all linkage technologies, certain risks to confidentiality persist, including the theoretical possibility of re-identification, particularly in scenarios with small k-anonymity sets.

Linkage accuracy depends heavily on the configuration of matching algorithms and the quality of input data. Although only a few false negative matches were identified in past projects (e.g., due to typographical errors in patient IDs), this highlights the importance of scenario-specific tuning of the linkage strategy. Since the EUPID Services support various PPRL algorithms, linkage accuracy has not been described here in a quantitative way. In a recent review, Tyagi & Willis (65) provided an overview of the accuracy of different algorithms, which can act as a reference for selecting the most suitable algorithm for specific applications.

Some components of the infrastructure, such as the Fair Data Point (FDP) interface and full Beacon 2.0 API support, are being implemented. Moreover, certain operational steps, such as pseudonym merging and re-identification, currently involve manual processing. While functional, this introduces inefficiencies and potential sources of human error that future automation efforts could mitigate.

Several areas of development are underway to further enhance the EUPID Services. Current research is exploring phonetic hashing techniques which are better suited for further languages, such as Metaphone 2 (66), which would support broader internationalization. Improvements in consent management—particularly in alignment with the Smart FOX project (62) and related works on dynamic consent, Common Conditions of Use elements and Digital Use Conditions (67, 68)—are expected to strengthen governance and user autonomy. In addition, we will continue to improve the usability of the EUPID Services for different user groups, including patients, e.g., based on mobile EUPID applications (69).

Within the European Joint Programme for Rare Diseases, a concept for integrating the EUPID Services into the EJP-RD/ERDERA Virtual Platform has been developed (54). Finalization of the respective FDP for metadata discovery and the Beacon-2-API for querying EUPID data are currently ongoing.

While the EUPID Services support linkage of patient identities, subsequent linkage of clinical data requires semantic interoperability of the clinical data. Therefore, clinical data standards such as ICD-11, SNOMED-CT or LOINC are recommended. Our data node technology (63) takes use of the OMOP Common Data Model (70), an open community data standard designed to standardise the structure and content of diverse observational healthcare data sources, enabling reliable cross-site analyses and collaborative research across observational clinical and administrative datasets.

Recently, multiple papers of the group of Han et al. have been published which focus on the application of multi-party computation and/or homomorphic encryption on PPRL (71–73). Ongoing research includes the extension of the EUPID framework with capabilities for homomorphic encryption and secure multi-party computation, either to avoid the exchange of hashed/encrypted/Bloom-filtered QIDs completely or to provide even more secure protocols for record linkage based on these techniques. Enhanced interoperability with other PPRL systems, improved statistical linkage services, and automated linkage quality assessments are key areas of focus. These enhancements will not only improve accuracy and usability but also better support secondary data use under the forthcoming EHDS framework.

## 5 Conclusion

PPRL is essential for enabling secure federated research infrastructures that support the primary and secondary use of health data. To maximise impact on initiatives like the EHDS, PPRL solutions must meet diverse, use case-specific requirements. The EUPID Services offer a configurable PPRL framework for healthcare and serve as a potential model for future implementations. Broad adoption of such services could enhance data-driven research and AI applications, ultimately advancing clinical innovation and reducing healthcare costs.

## Data Availability

The datasets presented in this article are not readily available due to privacy concerns—based on the core principle of the EUPID Services. Requests to access the datasets should be directed to Dieter Hayn, dieter.hayn@ait.ac.at.
